# What matters for drug delivery to tumor by nanoparticles: Gaining insights from PBPK/PD simulation of drug nanocrystals

**DOI:** 10.5599/admet.2415

**Published:** 2024-07-23

**Authors:** Shan Lu, Clairissa D. Corpstein, Kinam Park, Tonglei Li

**Affiliations:** 1 Department of Industrial & Physical Pharmacy, Purdue University, West Lafayette, Indiana, United States

**Keywords:** Murine tumor model, pharmacokinetic modeling, tumor accumulation, biodistribution, clinical trial

## Abstract

**Background and purpose:**

In our previous studies, drug nanocrystals were directly prepared by solution crystallization, possessing uniform particle size and morphology suitable for intravenous (IV) injection. These nanocrystals accumulated in a small percentage of their injected dose in tumor-bearing mice but showed similar anti-tumor effectiveness and much-reduced side effects compared with current commercial solubilized and encapsulated delivery systems.

**Experimental approach:**

In this study, we aimed to delineate possible controlling factors for the pharmacokinetics (PK) and biodistribution behaviors of paclitaxel (PTX) nanocrystals tested in mice by applying physiologically based pharmacokinetics (PBPK) modeling, coupled with pharmacodynamics (PD) simulation, to the data.

**Key Results:**

Our results show that clearance of the drug plays a significant, if not the most important, role in determining tissue distribution, including tumor accumulation of PTX nanocrystals. Surface treatment of drug nanocrystals with polymeric surfactants also appeared to affect PK profiles and PD outcomes. Importantly, when scaled to model human parameters, our PK/PD simulations suggest that drug distribution in humans, as opposed to animal models, was significantly influenced by tissue partitioning rather than drug clearance. This finding could facilitate the design and development of future drug delivery systems.

**Conclusion:**

Drug nanocrystals deposited in tissues, including tumors, could therefore act as depots, releasing the drug back into the circulation, possibly contributing to extended treatment, as well as any detrimental effects.

## Introduction

Over the last two decades, various nanoconstructs of chemotherapeutic agents have been fabricated and tested in tumor-bearing mice. In these systems, drug molecules are typically dispersed and encapsulated by polymeric materials such as so-called nanoparticles, often decorated physically or chemically with ligands aimed to bind to receptors overexpressed on tumor cells and subsequently reduce systemic toxicity. Most novel designs of nanoparticles, however, have not led to a satisfactory return in clinical applications commensurate with the extensive efforts, manpower, and research funding invested [1,2]. The impetus for nanoparticle-based cancer therapies has been rooted in the concept of enhanced permeation and retention (EPR) effect, which states that vasculatures in a solid tumor are “leaky,” allowing the preferred accumulation of nanoparticles in the tumor [3-5]. Underdeveloped lymphatic structures and lack of fluid drainage further signify this effect. The concept has led researchers to engineer a myriad of nanoparticle designs exhibiting unique physicochemical properties (size, shape, surface chemistry, *etc.*) targeted to a multitude of biological and pathological functions to improve drug efficacy [2,6]. While the underlying role of the nanoparticles in tumor targeting has been argued from both sides [7,8], one consensus among researchers in developing nanomedicines is that further understanding of in vivo behaviors of a nanoparticle system, including interactions with tissues and cells, is needed. Such knowledge of drug nanocrystals is scarce.

Our studies towards developing drug nanocrystals for chemotherapy are built upon the rationale that a poorly water-soluble drug can be delivered intravenously (IV) directly as solid, crystalline particles without using any solubilizing chemicals, as long as their particle size remains within a few hundred nanometers [9-15]. Uniformity in particle size and morphology is therefore crucial in maintaining physical stabilities and ensuring predictable drug release kinetics. Surface treatment of drug nanocrystals may be desired by the physical adhesion of polymeric surfactants to prolong systemic circulation. Nanocrystals are physically more stable than other nanoparticle formulations and can achieve nearly 100 % drug loading. We have mainly focused on testing bare and surface-treated paclitaxel (PTX) nanocrystals in murine tumor models [10,11]. In these studies, biodistribution and pharmacokinetics (PK) were determined by measuring drug concentrations in blood and major organs (including liver, heart, lung, spleen, and kidney) and in tumor. Antitumor efficacy and treatment toxicity were evaluated by monitoring tumor volume, body weight, and overall morbidity and compared to results of murine tumor models treated with conventional formulations (*i.e.* Taxol).

Our results indicate that the PTX nanocrystals physically treated by Pluronic® F-68 (triblock copolymer of poly(ethylene glycol), PEG, and poly(propylene glycol), PPG) achieved higher efficacy toward tumor growth inhibition and more significant drug accumulation in the tumor than bare PTX NCs [11]. Loss in body weight was generally lower in the nanocrystal-treated mice than in the Taxol-treated group, indicating less systemic toxicity elicited by the nanocrystals [10,11]. While the drug of Taxol was distributed extensively to all major organs upon IV administration, NCs were cleared rapidly from the blood circulation and primarily taken up by the liver, spleen, and lungs because of phagocytic sequestration. The F-68 treated NCs showed less uptake by the liver than bare NCs, though this was still significant compared to Taxol. Moreover, surface-treated NC accumulated more in the tumor than bare NC (2 *vs.* 1 % of injected dose, respectively). The finding suggests that the drastic increase in tumor accumulation of a nanoparticle delivery system, possibly due to the EPR effect, is not seen in the animal models, seemingly in agreement with other nanosystems [2]. Furthermore, the tumor accumulation of Taxol was on par with the NCs, possibly due to micellization of the drug, despite its biodistribution being completely different [16]. These studies raise an interesting and fundamental question of what factors determine the tissue distribution and tumor accumulation of a nanoparticle delivery system. The particle size and surface chemistry of a nanoparticle-based delivery system affect PK behaviors, but mechanistic interactions between nanoparticles and biological systems remain less studied. In particular, little is known about the in vivo fate of drug nanocrystals, which is complicated by the dynamic dissolution process of solid particles and the release of free drug molecules.

In the current study, we attempted to conduct PK modeling with a physiologically based pharmacokinetics (PBPK) method, coupled with pharmacodynamics (PD) simulations, to explore possible factors determining drug nanocrystals' biodistribution and treatment efficacy. By fitting these models to our in vivo data obtained in animals, it is possible to delineate various factors on transport kinetics and in vivo performance. The PBPK model has been established as a prediction framework for quantitatively describing drug absorption, distribution, metabolism, and elimination kinetics by considering the kinetics of mass balance and drug transport in each organ [17,18]. PD modeling quantifies efficacy kinetics according to the systemic and local exposures of drug treatment. A sensitivity analysis was performed to evaluate the relationship among various PBPK parameters and identify significant factors. Additionally, the modeling and simulation were scaled to human models to shed light on the limitations of using animal models for testing drug delivery systems.

## Methodology

### PBPK Modeling

**Figure 1.**
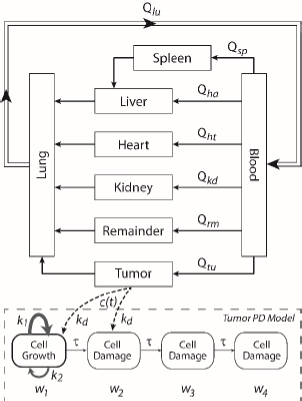
Scheme of whole-body PBPK-PD model.

A whole-body physiologically based pharmacokinetics (PBPK) model was constructed (Figure 1). It included several major organ compartments, including lungs, blood, heart, liver, spleen, intestine, kidney, and tumor. All other tissues were lumped into the Remainder compartment. Drug transport in each compartment was assumed to be homogeneously distributed and perfusion-limited modeling was utilized [19,20].

Following the mass balance through all the compartments, a general differential equation (1) was used to quantify drug concentration in a compartment:



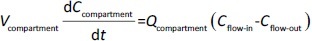

[1]


Where *C*_compartment_ / M is drug concentration, *V*_compartment_ / L is volume, and *Q*_compartment_ / L h^-1^is blood flow rate in the particular organ or compartment. *C*_flow-in_ represents the drug concentration in the blood flowing into the compartment and *C*_flow-out_ represents that flowing out. In the following equations, subscripts are used to denote respective organs, bl for blood, lu for lung, li for liver (in particular, *Q*_li_ denotes hepatic portal vein flow rate and *Q*_he_ *Q*_he_ hepatic artery flow rate), ht for heart, kd for kidney, tu for tumor, sp for spleen, and rm for remainder. Assuming drug diffusion in the tissue is not the rate-limiting step, the drug concentration in each organ can be described by drug partitioning between the blood and organ as quantified by the partition coefficient, *K*_compartment_, which is unitless. PTX elimination mainly occurs in liver [21], which is assumed to be a linear process described by *CL*_liv_ . Each compartment is then described as by the following equations (2) to (9).



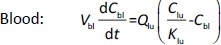

[2]




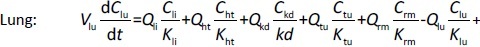

[3]




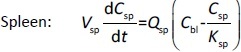

[4]




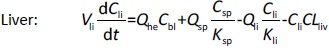

[5]




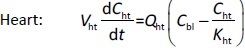

[6]




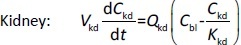

[7]




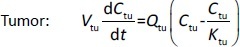

[8]




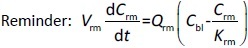

[9]


The multiple terms in the lung equation account for the venous flow-in from major organs and the arterial flow-out (Figure 1). The liver equation considers the flow-in from the spleen and hepatic clearance of the drug. Table 1 lists the physiological parameters used in the equations; the blood flow rates were obtained from the literature [22,23] and the organ volume were measured in our animal studies used to evaluate PTX NCs. Organ volumes were determined by organ weights with the tissue density of 1.18 g/ml [24]. Partition coefficients and the liver clearance rate were determined by data fitting of the PBPK model against our experimental values using nonlinear least squares (NLS)[25]. The PK parameters in the above questions, including partition coefficients, are adjusted and optimized during the NLS process to minimize the resident errors between the experimental data and model-predicted values of drug concentrations. The model was fitted with a tolerance of 10^-8^. All calculations were done by MATLAB (version R2018a [26]).

**Table 1.** Physiological parameters used for mice.

Tissue	Plasma flow, L/h	Organ volume, L
Blood	0.48^ǂ^	0.0017^ǂ^
Liver	0.108^ǂ^	0.0015*
Spleen	0.0054^ǂ^	0.0000847*
Heart	0.0168^ǂ^	0.0000678*
Lung	0.48^ǂ^	0.00010*
Kidney	0.078^ǂ^	0.00027*
Tumor	0.00102^ǂ^	0.0016*
Remainder	0.27618	0.013*
Hepatic flow rate	0.021^ǂ^	

^ǂ^Plasma flow and blood volume were obtained from the literature [22,23].

*Organ volume was calculated with average measurement reported in our previous study [11].

### PD Modeling

A transit compartmental PD model was coupled with the drug exposure kinetics in the tumor that is calculated by PBPK (Figure 1) [27-29]. In the early stage of cell proliferation, tumor growth is treated exponentially. Angiogenesis and nutrient depletion will impede the process at the later stage, resulting in the linear growth of tumor [30]. To account for the lapse between drug exposure and cell apoptosis, three transit compartments are utilized in the PD model (Equation (10)):



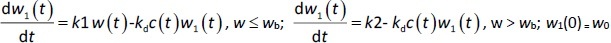

[10]


where *w*_1_ / g describes the tumor weight with the initial exponential growth characterized by the rate, *k*_1_, followed by the linear growth characterized by the rate, *k*_2_, and *w*_b_ is the weight cutoff above which the tumor growth switches from the exponential to a linear model. *w*_0_ represents the initial tumor weight, and *k*_d_ denotes the tumor cell elimination rate by the drug with *c* denoting the drug concentration, which is calculated by the PK model. Moreover, compartments *w*_2_ and *w*_3_ are introduced to compensate for the lapse of tumor growth upon drug exposure, Equations (11) to (15):



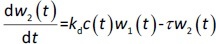

[11]




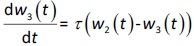

[12]




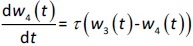

[13]




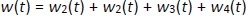

[14]




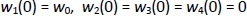

[15]


where *w* is the total tumor weight and is the transit parameter to define the treatment lapse. In this study, NLS was first applied to fit the model against experimental data of tumor weight of mice treated with saline and obtain the tumor growth factors (*k* values and ).

### Sensitivity analysis and scaling to human

To quantitatively assess the relative importance of each PBPK parameter that was obtained from NLS fitting on the PK and biodistribution of drug treatment, a sensitivity analysis was conducted. According to our PBPK model, drug distribution and accumulation mainly depend on blood flow rate, tissue partition coefficient, and renal clearance. Derivative-based local sensitivity was performed by applying the one-at-a-time (OAT) technique to evaluate the effect of one parameter on drug concentrations in organs while keeping other parameters fixed [31,32]. Specifically, sensitivity is calculated by taking the derivative of a particular output (*e.g.* drug concentration in the liver) over the parameter of interest (*e.g.* clearance), Equation (16):



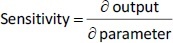

[16]


As the output (*i.e.* concentration) is a function of time, a calculated sensitivity is then integrated over the treatment time. All the sensitivity values are assembled into a final sensitivity matrix for reporting with the output properties (*i.e.* concentrations in all respective organs) and PBPK parameters marked.

Finally, the PBPK model was scaled to humans, and PK simulation was conducted. We assumed that organ partitioning of PTX NCs is similar between mice and humans. To do this, physiological data was replaced by human values taken from literature [22,33], shown in Table 2. The clearance of the drug was also replaced with that in humans [34]. Note that the tumor data was of breast tumor, and its plasma flow was determined by macroscopic approaches [35].

**Table 2.** Physiological parameters used for humans (70 Kg).

Tissue	Plasma flow, L/h	Organ volume, L
Blood	336	5.2^ǂ^
Liver	87	1.69^ǂ^
Spleen	4.62	0.192^ǂ^
Heart	14.4	0.31^ǂ^
Lung	336	1.17^ǂ^
Kidney	74.4	0.28^ǂ^
Tumor	3.2	0.00419^ǂ^
Remainder	157	55.9
Hepatic flow rate	82.38	

^ǂ^Organ volume was obtained from the literature [22, 33].

## Results and discussion

### PBPK Modeling

**Figure 2.**
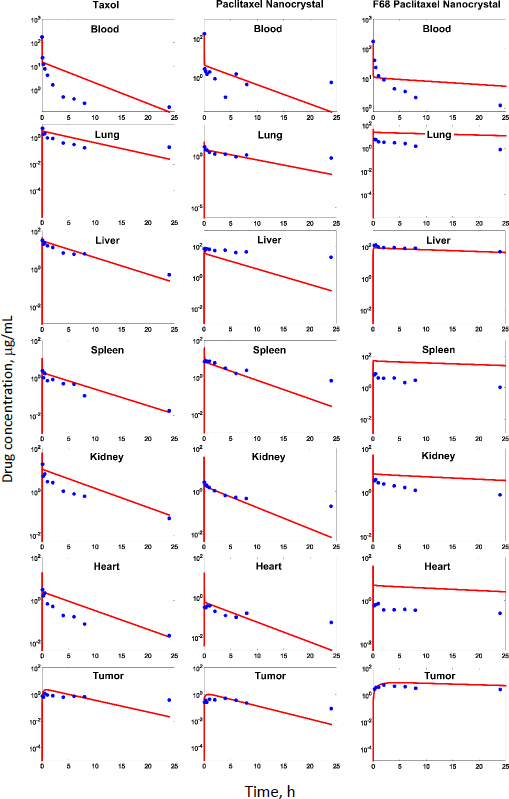
Concentration of three PTX formulations following 20 mg/kg IV injection with respectively fitted lines (red).

In our previous studies with paclitaxel (PTX) nanocrystals, PK and PD kinetics were determined in tumor-bearing mice [10,11]. The murine tumor model was established by subcutaneous injection of 3×10^6^ HT-29 cells under the right arm and 3×10^6^ KB cells under the left arm of female nude outbred mice. Taxol, pure PTX nanocrystals and PTX nanocrystal surface-coated with F68 were administered intravenously once at an equivalent dose of 20 mg/kg via the tail vein. Biodistribution and pharmacokinetics were measured. By fitting the data with our PBPK model, partition coefficients in the major organs and the liver clearance were obtained (Table 3). The fit lines are shown in Figure 2.

**Table 3.** Estimated pharmacokinetic parameters of three formulations of paclitaxel.

Partition coefficient	Taxol	CV*, %	PTX-NC	CV*, %	F68-PTX-NC	CV*, %
*K*_li_	2.15	23.64	10.99	19.98	6.92	7.95
*K*_sp_	0.13	9.79	1.04	35.63	0.32	15.61
*K*_ht_	0.17	9.86	0.26	7.12	0.036	9.11
*K*_kd_	0.75	12.06	0.41	17.02	0.18	10.67
*K*_lu_	0.21	9.98	0.58	20.43	0.25	0.0012
*K*_tu_	0.27	14.35	0.28	19.00	0.65	23.43
Clearance, L/h	0.0023	0.06	0.0026	0.0052	0.000091	0.00083

*Coefficient of variation

Among all compartments, the partition coefficient of the liver is the highest for all three delivery forms. Compared to the conventional Taxol delivery system, the partition coefficients for the nanocrystal formulation increased as such: liver by 5-fold, spleen by 8-fold, and lung by 2-fold. This is likely due to the MPS sequestration, with the liver being the major organ for removing nanoparticles from blood circulation. For Taxol, the kidney had the second highest partition coefficient of 0.75 (unitless) after the liver; the number decreased to 0.41 for the nanocrystal formulation. With the increased liver uptake and decreased renal clearance, the liver could be considered a major depot site for drug nanocrystals. Moreover, surface treatment by F68 reduced the liver partition coefficient from 10.99 to 6.92, which was still 3.3 folds greater than Taxol's. F68 coating minimized the liver uptake and, compared with the other two formulations, significantly lowered clearance, suggesting prolonged blood circulation. The surface treatment also eased the drug tissue affinity in the spleen, heart, kidney, and lung, helping to lighten toxic effects in these organs and enhancing drug circulation. In the tumor, the partition coefficients of Taxol, PTX-NC, and F68-PTX-NC are 0.27, 0.28, and 0.65, respectively. The surface treatment increased the tumor partition (by 2.5 folds), supporting our experimental observation in animals [10,11].

While keeping the partition coefficients and parameters fixed, we ran PK simulations with several different values of the tumor partition coefficient. Table 4 shows the simulation results with the partition coefficient ranging from the smallest value of 0.27, which was of Taxol, to a more significant, hypothetical number of 0.87 for all three formulations. Both maximal concentration (*C*_max_) and area under curve (AUC) values in tumor became larger as the tumor partition coefficient (*K*_partittion_) increased. The *C*_max_ increased about two times and AUC about three to four times due to the increase in tumor partition. Thus, given the same dose, the higher the tumor partition coefficient, the more drug accumulation in the organ, resulting in better treatment efficacy. F68-PTX-NC showed the highest PK performance in the tumor compartment because of decreased tissue affinities with the surface treatment among the three delivery systems.

**Table 4.** Simulation results with different tumor partition coefficients.

Tumor *K*_partittion_	AUC Taxol	*C*_max Taxol_ / μg ml	AUC PTX-NC	*C*_max PTX-NC_ / μg ml	AUC F68-PTX	*C*_max F68-PTX_ / μg ml
0.28	8.75	2.78	5.31	1.91	55.16	2.77
0.43	13.40	3.74	8.13	2.54	83.23	4.14
0.57	18.048	4.53	10.95	3.057	110.56	5.47
0.72	22.70	5.20	13.78	3.49	137.092	6.74
0.87	27.35	5.79	16.60	3.87	162.84	7.96

### PD Modeling

**Figure 3.**
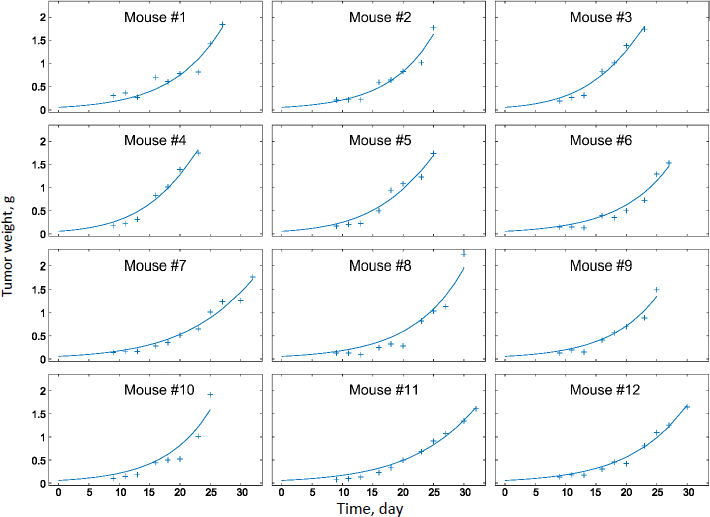
Tumor growth of 12 individual mice treated with saline (control group)

Experimental measurements of tumor growth from our earlier studies were used to establish the PD model. Values of the control treatment group were first fitted in order to obtain the growth rate parameters of the tumor (*k*_0_ and *k*_1_ in Eq. 11). Figure 3 shows the experimental data and predicted trends in tumor growth up to 23 days of individual mice injected with saline solution. The tumor growth model was able to describe the experimental data well. The average tumor linear growth factor *k*_1_ and exponential growth factor *k*_2_ were 0.17 (± 0.11) and 0.51 (± 0.56) grams per day, respectively. The growth factors were then applied to analyze *k*_d_ and of the drug treatment by Taxol and PTX NCs.

The tumor treatment parameters were obtained by fitting the PD model against the experimental data with the availability of *k*_1_ and *k*_2_. The transit parameter τ that characterizes the kinetics of cell damage are 0.93, 0.96, and 0.95 day^-1^ for Taxol, PTX-NC, and F68-PTX-NC, respectively. The parameter to quantify drug efficacy, *k*_d_, is 0.0039, 0.0037, and 0.0038 mL ng^-1^ day^-1^ of each respective treatment. The minor differences in the parameters among the drug delivery systems indicate similar anti-tumor activities. This could be understandable as the delivery systems containing the same drug, PTX, and the nanocrystal delivery systems do not alter the pharmacodynamics behavior. It also suggests that a nanocrystal has no antitumor effect; it is released or dissolved, whereby free drug molecules elicit the antitumor function.

### PBPK/PD Simulation in humans

**Figure 4.**
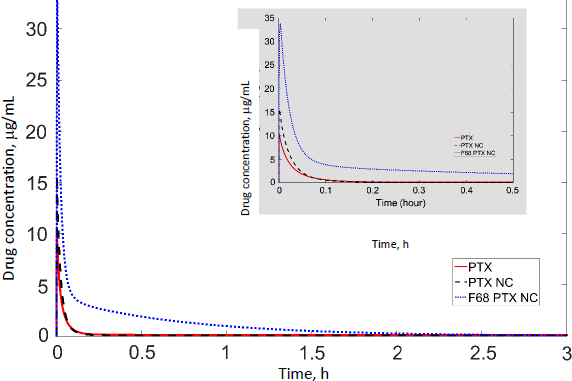
PK simulation of the tumor compartment in a human using three PTX formulations (20 mg/kg) over 3 h. The inset shows a simulation to 0.5 h.

We scaled the PBPK model to humans and conducted PK simulations under hypothetical conditions. The same partition coefficients determined by data fitting of the murine model were utilized; physiological parameters (Table 2) and the clearance value of PTX (25.81) in humans were taken from the literature [34]. The simulation results of the tumor compartment are shown in Figure 4. The initial spikes of concentration were likely caused by the mathematical model originally generated by data fitting of experimental results obtained in mice. The remaining trends appear more trustworthy; thus, only the AUC should be used to interpret the simulation results. The AUC values of drug accumulation in tumors are 1.54, 1.16, and 3.52 μg/ml·h of Taxol, PTX-NC, and F68-PTX-NC, respectively. The surface treatment seems to at least double the drug accumulation in tumors compared with the solubilized formulation or pure NCs. Relative to the total injected dose (316 mg for 70 kg adult with body surface area 1.7 m^2^), the tumor accumulation was 0.29, 0.44, and 0.95 %. The trend is similar to that in mice (0.62, 0.26, and 2.92 %). The further increase in mice for F68-coated NC resulted from the decreased clearance. Comparison of other organs indicates different trends between mice and humans (Table 5). Each value in the tablet is relative to the total injected dose, equivalent to the accumulated concentration in blood (*i.e.* 100 %).

**Table 5.** Comparison of tissue partition between mice and humans by three different treatments relative to the total injected dose.

	Relative mass to that in blood, %					
	Mice			Human		
	Taxol	PTX-NC	F68-PTX	Taxol	PTX-NC	F68-PTX
Lung	0.16	0.26	0.19	1.05	4.12	1.61
Blood	100	100	100	100	100	100
Heart	0.07	0.01	0.02	0.66	0.57	0.19
Kidney	1.69	0.24	0.34	2.81	1.73	0.85
Liver	15.89	35.16	64.38	14.26	17.67	18.55
Spleen	0.07	0.23	0.22	0.29	1.77	3.28
Tumor	0.62	0.26	2.92	0.29	0.44	0.95

**Figure 5.**
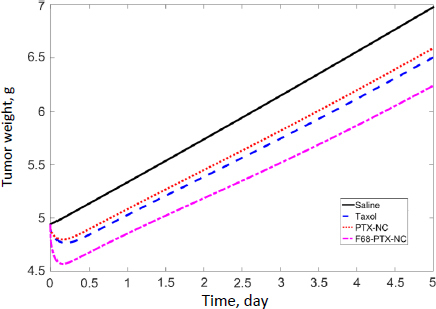
Human PD simulation of three paclitaxel drug formulations.

We further simulated PD in humans based on the tumor exposure predicted by the PBPK discussed above. Tumor growth parameters were taken from our mouse model. Data from human breast tumors were simulated with the blood flow rate and tumor volume taken from the literature (3.2 L/h and 0.00419 L; Table 2). Note that, in our tumor-bearing mice (as well as numerous tumor murine models reported in the literature), the xenografted tumor is significantly larger relative to body weight (6 %) and, therefore, not scalable in humans (0.007 %; see later discussion). Figure 5 shows tumor growth inhibition by these treatments was evident within the first few hours (3.6, 2.9, and 7.5 % with Taxol, PTX-NC, and F-68-PTX-NC, respectively). At day 5, human breast tumor growth without treatment increased by 41.2 %, while the increases were 31.7, 33.4, 26.3 % of Taxol, PTX-NC, and F68-PTX-NC, respectively. In comparison, the tumor growth during a 5-day span in mice without treatment was 270 and 46-79 % with the treatments. The results show a considerable difference in tumor growth between mice and humans without treatment. Even with the treatment, the difference is still outstanding. The simulation also indicates that surface coating of NCs can positively inhibit tumor growth, agreeing with the biodistribution simulation (Figure 4).

### Sensitivity analysis and human-scaling simulation

**Figure 6.**
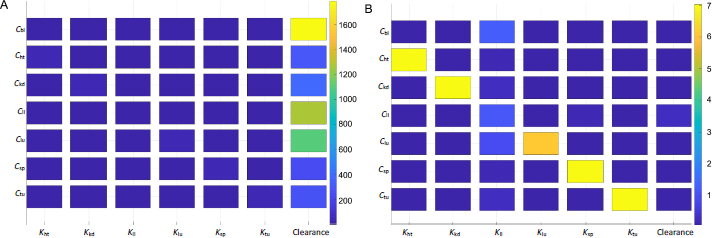
Heat maps or sensitivity matrices of seven organ compartments with eight PK parameters, including the partition coefficient of each compartment and liver clearance in mice (A) and in humans (B). PK parameters are shown along the x-axis and drug concentrations in the organs are shown along the y-axis. Values are sensitivities accumulated over 3 hr.

Sensitivity analyses were conducted to examine the impact of individual PK parameters on the biodistribution kinetics in mice and humans. Figure 6 shows the two sensitivity matrices calculated for mice and humans. The data shown here was generated based on simulations of Taxol (considering the clearance data taken from the study of Taxol), but the data of the other two NC formulations (not shown here) yield the same trend and conclusion.

The heat map indicates intercorrelations between PBPK parameters and drug concentrations in the organs and tumor. For the drug concentration in a particular organ (*i.e.* the y-axis), the figure ranks the significance of impact by the respective partition coefficient and hepatic clearance (x-axis). Each box's color marks the sensitivity values of a particular derivative of organ concentration over partition coefficient or clearance, integrated over a period of time (*e.g.* 3 h used in Figure 6). The sensitivity map calculated from the mice PK model (Figure 6A) indicates that the hepatic clearance of PTX is the dominant factor in determining drug depositions in all organs and the tumor. The plasma concentration of the drug is affected mainly by the hepatic clearance. The next organ affected by the clearance is the liver, followed by the lung. From the mathematic and modeling perspective, the drug concentration accumulated in an organ is positively affected by the blood flow rate to the organ and negatively by the organ volume (or weight). The lung has the highest blood flow rate, followed by the liver. This can be further illustrated by comparing drug accumulations in tumors and the liver. The tumor has a comparable organ volume to the liver or blood, but its flow rate is only a fraction of that in the liver or blood (about 0.9% or 0.2%; Table 1). Besides the phagocytosis of drug nanocrystals by the MPS (indicated by the liver partition coefficient), the much-dwarfed systemic circulation into the tumor significantly limits drug deposition. Nonetheless, despite the liver partition being the largest among all partition coefficients (Table 3), it is the systemic clearance by the liver that exaggerates the liver partition in determining local drug distributions to other organs and the tumor.

Figure 6B shows the sensitivity matrix in humans. The same partition coefficients determined from the mice model were utilized but with human physiological conditions (Table 2). The clearance was taken from human experimental data for paclitaxel [34]. The overall result is different from that in mice. The partition coefficients of each organ and tumor have the most relevant impact on drug accumulation. On the other hand, the hepatic clearance is not significant. The liver partition coefficient is the most influential parameter for blood plasma instead of clearance. Ostensibly, the sensitivity data indicate that instead of systemic elimination, drug distribution to organs contributes the most to drug disposition. The variations in physiological and anatomical conditions primarily cause the difference in biodistribution between mice and humans. In humans, the cardiac flow rate (*Q*_lu_) is about four times greater than that in the liver; the blood volume is also 3-4 times higher (Table 2). In mice, however, even though the liver blood flow rate is about four times smaller than that in blood, the liver volume is similar to the blood volume. Given the same drug partition coefficients used in both mice and human models, such differences in blood flow and organ volume explain drastically different PK behaviors of drug nanocrystals between the two biological systems. This also implies that hepatic uptake and clearance are more significant in rodents, including mice than in humans. From an evolutionary perspective, it could make more sense that humans prepare and cook their food while animals digest raw organics. A stronger MPS would protect the body from exogenous particles and toxins absorbed into the systemic circulation. Additionally, it is noted that the scales are significantly different in the sensitivity maps between mice and humans, as shown in Figure 6.

A sensitivity value is much higher in mice than humans at any time point of simulation – mathematically, a direct result of the relative ratio between blood flow rate over organ volume (or weight) being more significant in mice than humans.

**Figure 7.**
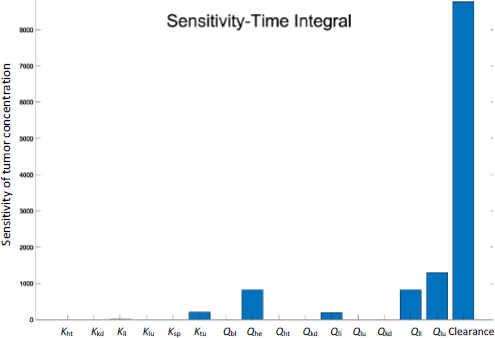
Sensitivity analysis of drug concentration in tumor with respect to PK parameters in mice. Values are sensitivity values accumulated over 24 h.

Figure 7 shows the time-integrated sensitivity values in mouse tumors over 24 hours. Again, it is shown that the hepatic clearance has the most significant impact on the drug accumulation, four times greater than the next contribution, tumor blood flow rate, followed by blood flow rates through the hepatic artery, spleen, and liver. While the blood flow rate and organ volume are both small in the spleen (Table 1), drug transport and deposition in the spleen directly contribute to the drug concentration in the liver (Eq. 5). The Tumor partition coefficient ranks as the fourth factor to alter the drug accumulation. This suggests that even with the so-called active targeting moieties on the surface of nanoparticles, the formulation strategy would have a minimal effect in tumor accumulation. Moreover, given that tumor blood flow only accounts for 0.2% of the total blood output, any possible impact by the EPR effect is very limited in varying the drug accumulation in the tumor relative to the total amount of injected dose. In comparison, blood flow in the liver and kidney accounts for more than 22.5 and 16.25 % of the cardiac output, respectively (Table 1).

Our analyses suggest that in mice when given intravenously, drug nanocrystals are mainly taken up by the liver; tissue distribution and tumor accumulation of the drug are subsequently determined by how fast the drug is eliminated from the system. However, tissue distribution in humans is likely driven by the systemic circulation and the organ affinity of the drug (delivery system). In humans, the drug has a greater tendency to be deposited in tissues and gradually released back into the systemic circulation (blood and lymph). With this regard, having a drug delivery system circulating long enough in the blood (for example, by putting biocompatible surfactants on nanoparticle surfaces) could significantly enhance tissue distribution and tumor accumulation in mice. Conversely, having such surface treatment or not solely to improve the blood circulation might make little distinction in humans. Although the blood concentration of PTX doubled (Figure 4), this may result in higher tissue partitioning of the NC surface coating. From the systemic perspective, the PK difference implies that treatment efficacy and PK of a drug delivery system tested in murine models may not directly extend to humans. As indicated by a literature report, two different formulations of PTX were seen to have opposite rankings in anticancer effects in mice vs. human clinical trials [36].

## Conclusions

In this study, we applied PBPK and PD modeling to analyze animal test results of PTX nanocrystals. Sensitivity analyses were conducted of the PK parameters derived from the modeling study, and scaling the PK/PD models to humans was attempted. The modeling results shed some light on tumor targeting by nanoparticular systems, especially drug nanocrystals. Because of the deficient blood supply to tumors, drug accumulation is inherently limited. In mice, drug clearance of nanocrystals by the liver is the most critical factor in determining the biodistribution and PK in the body. Surface treatment of nanoparticles to prolong their blood circulation will likely have a positive effect on mitigating the clearance and enhancing tumor accumulation. The impact of leaky vasculatures and deficient lymphatic drainage in tumors (i.e., so-called EPR effect) on drug deposition to the tumor, however, is minimal. In humans, it is the tissue partitioning, not the systemic clearance, of the drug that determines drug deposition in respective organs. Tissue partitioning is affected by the blood supply, vasculature fenestration, and drug binding with extracellular proteins and other molecules. Consequently, surface treatment to enhance blood circulation may have a limited effect on tumor accumulation in humans. On the other hand, leaky blood vessels could increase drug deposition in tumors. All in all, while mouse models essentially showed liver uptake of NC and improved tumor targeting of NC through F68 coating, PK behaviors in humans of this drug delivery system may be drastically different. It seems plausible that even without surface-bound polymers, drug nanocrystals could still have satisfactory antitumor efficacy as the drug may be available in the circulation and target site from those deposited in tissues and organs.

Caution needs to be taken when generalizing these conclusions. Our extrapolation to humans is a mathematical exercise based on human physiological parameters with drug partitioning to each organ derived from modeling experimental data from mice (albeit the dose and clearance taken from human trials). Interpretation of the simulation data merely highlights the impact on PK and tumor targeting, resulting from the differences in physiology between mice and humans. Future studies of modeling clinical trial data are necessary.
